# Raptor migration in an oceanic flyway: wind and geography shape the migratory route of grey-faced buzzards in East Asia

**DOI:** 10.1098/rsos.171555

**Published:** 2018-03-07

**Authors:** Elham Nourani, Kamran Safi, Noriyuki M. Yamaguchi, Hiroyoshi Higuchi

**Affiliations:** 1 Laboratory of Animal Ecology, Graduate School of Fisheries and Environmental Sciences, Nagasaki University, Nagasaki, Japan; 2 Max Planck Institute for Ornithology, Radolfzell, Germany; 3 University of Konstanz, Konstanz, Germany; 4 Research and Education Center for Natural Sciences, Keio University, Yokohama, Japan

**Keywords:** *Butastur indicus*, step selection function, water-crossing, wind support, least-cost path, East-Asian oceanic flyway

## Abstract

Flapping flight is relatively costly for soaring birds such as raptors. To avoid costly flight, migrating raptors generally avoid flying over water. As a result, all but one of the global raptor migration flyways are largely over land. The East Asian oceanic flyway for raptors is the exception. Raptor species using this flyway migrate by island-hopping, flying over open ocean for distances of up to 300 km between islands. We used satellite telemetry data for grey-faced buzzards *Butastur indicus*, a species that dominates the southern part of the flyway, to investigate the geographical and atmospheric factors responsible for the suitability of this flyway for raptor migration. Using a combination of least-cost path analysis and a step selection function, we found that the occurrence of numerous islands and also suitable wind support along the oceanic flyway are responsible for route selection in grey-faced buzzards. These results confirm the role of islands, but also wind, in shaping the East Asian oceanic flyway of long-distance raptor migration.

## 1. Introduction

Many raptor species accomplish their long-distance migrations predominantly by soaring on thermals and orographic updrafts [1–3]. They are generally reluctant to fly over water bodies, where thermals are weak or absent in most latitudinal zones and where powered flight is required [1–4]. Because of these adaptations [5], raptors migrate along well-established flyways that occur mostly overland and circumnavigate water bodies or converge on narrow land bridges or short water-crossings where overwater flight is inevitable [1,6,7].

The East Asian oceanic flyway for raptors (hereafter the oceanic flyway) is an exception. This largely overwater flyway stretches from northeastern Siberia to Southeast Asia and is dominated by the Chinese sparrowhawk *Accipiter soloensis* and the grey-faced buzzard *Butastur indicus* [1,8,9]. It is assumed that the numerous islands that occur along this flyway provide an opportunity for migration by island-hopping. However, the long distances of up to 300 km between the islands, which is more than distances commonly covered by raptors over water along other flyways, indicate that additional factors, such as atmospheric currents, facilitate raptor migration.

Water-crossing behaviour in raptors around the world is attributed to wind support [10–12]. In the oceanic flyway, sea thermals might also be responsible for long water-crossings [3,13] as the southern part of the flyway falls within the trade-wind zone (i.e. 5° to 30° north and south of the equator), where thermals develop over the sea [14].

Here we investigate the influence of islands and atmospheric variables in shaping the oceanic flyway by using satellite telemetry data collected for grey-faced buzzards migrating from breeding areas in Japan's Kyushu Island to wintering grounds in the Philippines.

## 2. Methods

### 2.1. Satellite-tracking

**Figure 1.**
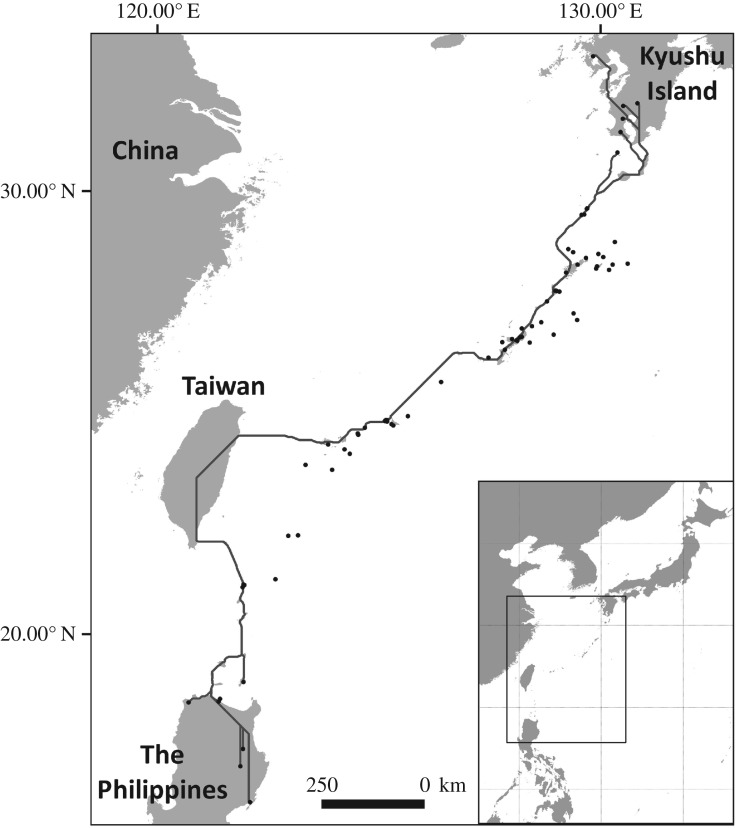
The least-cost route generated by minimizing water-crossing for migration of grey-faced buzzards from Kyushu Island to the Philippines (thick line). Note that this optimal route deviates from the observed trajectories (black dots).

Various populations of grey-faced buzzards breed in different parts of Japan and winter in Japan's Ryukyu Islands or the Philippines [8]. We focused only on the population that winters in the Philippines, as these individuals use a larger part of the oceanic flyway and perform long water-crossings. Data were obtained from tracking data collected in autumn (October) 2009 for grey-faced buzzards breeding in Kyushu Island of Japan (figure 1). The birds were captured in Itoshima, Fukuoka Prefecture in spring (May and June) 2009 using clap nets with live caged mice as bait. The trap was activated and flipped over the bird as the bird attacked the mouse cage. The mice were not harmed in the process. The birds were fitted with 12-gram solar-powered Platform Transmitter Terminals (PTTs; North Star Science and Technology, Inc., VA, USA), weighing less than 4% of their body weight. The PTTs were programmed to transmit for 14 or 8 h followed by 14 or 23-h periods without transmission, respectively. Data recorded in local daylight hours were used in this study.

### 2.2. Least-cost path analysis

By assuming that minimizing overwater flight results in less energetically costly migration, we generated an optimal route to connect the breeding and wintering grounds through the islands, while minimizing water-crossing. We used the inverse of a distance to coast layer as a conductance surface to find the shortest path (i.e. the least-cost path), in gdistance package [15] in R environment [16]. For each trajectory, the last point in mainland Japan and the first point in the Philippines were considered the starting and end points, respectively.

### 2.3. Step selection function

#### 2.3.1. Generating alternative steps

We modelled migratory route selection of the grey-faced buzzards using a step selection function [17]. In this method, the straight-line segment connecting successive tracking points is considered as one step (i.e. observed step) and a number of alternative steps from the same starting point is generated. We compared the observed and alternative steps based on a number of geographical and atmospheric variables using a conditional logistic regression approach, which allows taking into account stratification in the analysis [18].

We reduced autocorrelation by making sure that the consecutive data points used for the analysis were at least one hour apart. For each observed step (*n* = 61), we generated 100 random steps (see electronic supplementary material, S1). Length and turning angle of random steps were drawn from the frequency distribution of those of the observed steps.

#### 2.3.2. Annotation of steps with environmental data

All observed and random steps were annotated using the Env-DATA track annotation service in Movebank (https://www.movebank.org/), using bilinear interpolation. Variables included distance to coast, eastward and northward components of the wind, cloud cover, relative humidity to represent possibility of rain, and boundary layer height as a proxy for convective conditions. Atmospheric data were obtained from European Centre for Medium-range Weather Forecasts (ECMWF). Because the flight height of the birds was not known, pressure-level data were obtained at 900 mb level, corresponding to the average boundary layer height over the study area (*ca* 1000 m). We calculated wind support and crosswind along each step, where wind support was the length of the wind vector in the direction of the bird's heading and crosswind the length of the wind vector perpendicular to the movement direction. We estimated heading as the angle from the starting point to the endpoint of each step.

#### 2.3.3. Statistical analysis

All variables were checked for collinearity (|*r*| > 0.7) before analysis. Conditional logistic regression was carried out on scaled variables with the survival package [19] in R environment [16]. Groups of matched observed and alternative steps were entered as strata in the model.

We built models without interaction terms and with two-way interaction terms for distance to coast and wind support and crosswind respectively. We used backward stepwise model selection by removing variables that were non-significant in model building. The minimum adequate model was considered as the model with the lowest AIC (Akaike's information criterion) [20]. We then evaluated the performance of the best model by calculating the proportion of correct choices made by the model as a consequence of the fitted effects of the variables used. To do this, we used a *k*-fold cross-validation method suitable for the case--control design of the study [20,21] with 100 repetitions. To determine variable importance, we used a randomization procedure with ten permutations [22,23].

## 3. Results

The optimal route based on the least-coast path analysis connected Japan's Kyushu Island to the Philippines by passing through the Ryukyu Islands and Taiwan (figure 1). This route deviated from the observed trajectories. Particularly, unlike what the optimal route suggested, the tracked birds did not go through Taiwan to reach the Philippines.

### 3.1. Step selection function

Since variables did not show high collinearity, all were retained in the model building process. In the final model, with the lowest AIC with a delta AIC of 1.7 compared to the next best models (tables 1 and 2), distance to coast, wind support, crosswind and the interaction between distance to coast and wind support had a significant influence on route selection. Distance to coast was the most important variable describing route selection of grey-faced buzzards (relative importance: 0.44), followed by wind support (relative importance: 0.29) and crosswind (relative importance: 0.04). Cross-validation indicated that the model was a useful predictor of route selection in grey-faced buzzards (*k*-fold (*r*'s and ranges): observed, 0.40 (0.25–0.50) and expected by chance, 0.08 (0.00–0.17)).

**Table 1.** Results of the step selection function. Coefficients are shown for scaled variables used in the minimum adequate model (see table 2 for model comparison).

covariate	*β* ± s.e.	*p*
distance to coast	−1.36 ± 0.15	0.000
wind support	1.15 ± 0.34	0.001
crosswind	−0.41 ± 0.2	0.037
distance to coast * wind support	0.29 ± 0.15	0.049

**Table 2.** Model comparison using AICs in a stepwise backward selection approach. Coefficients are the results of modelling with scaled variables. Model 1 was built with no interaction terms. Models 2–6 included interaction terms and insignificant variables were removed one by one in a backward selection approach. Model 6 was considered the best model because it yielded the lowest AIC.

	coefficients									
model	distance to coast	wind support	crosswind	boundary layer height	cloud cover	relative humidity	distance to coast * crosswind	distance to coast * wind support	AIC	ΔAIC
1	−1.262	0.763	−0.354	0.035	0.010	0.085	n.a.	n.a.	355.199	—
2	−1.390	1.187	−0.525	0.039	0.067	0.069	−0.084	0.313	355.062	—
3	−1.392	1.191	−0.526	0.033	0.089	n.a.	−0.083	0.314	353.122	1.940
4	−1.389	1.182	−0.510	0.020	n.a.	n.a.	−0.074	0.310	351.245	1.877
5	−1.386	1.185	−0.511	n.a.	n.a.	n.a.	−0.074	0.310	349.253	1.993
6	−1.359	1.147	−0.410	n.a.	n.a.	n.a.	n.a.	0.290	347.5313	1.722

## 4. Discussion

The oceanic flyway comprises numerous islands that, as our results suggest, significantly contribute to its suitability for raptor migration. Migratory birds can use prominent geographical formations such as islands as leading lines for navigation purposes [1]. The role of islands in facilitating raptor water-crossing has been shown for honey buzzards *Pernis apivorus* migrating over the central Mediterranean. Honey buzzards crossing the Mediterranean between Italy and Tunisia use islands for orientation and wind drift compensation, and as sources of thermal uplift [24,25]. Moreover, Agostini *et al.* [24] suggest that breaking up a long sea-crossing into smaller sections by exploiting islands reduces the risk of encountering unpredictable weather changes that can interrupt non-stop flight over water.

The least-cost path that we constructed by minimizing water-crossing was not an exact representation of the birds' observed trajectories. Although the migratory routes and flyways of soaring migrants commonly deviate from the shortest route to save energy ([26]; cf. [10]), the autumn trajectories of grey-faced buzzards followed a shorter and more direct path than the least-cost route. This choice of route allowed the birds to reduce their overall migration duration, but would expose them to long non-stop overwater flight, e.g. by refraining from migrating via Taiwan. However, long sea-crossing can be energetically less expensive under suitable wind conditions [25]. The high importance of wind support detected by our step selection function suggests that this was probably true for grey-faced buzzards. Whether sea thermals also contributed to reducing the costs of overwater flight remains unknown, as the coarse resolution of our tracking data was not suitable for identifying the small-scale response of the birds to updraft conditions.

Raptors using the oceanic flyway show exceptional migratory behaviour and adaptations, yet it is one of the least studied migration systems in the world. Our study is a contribution to understanding the geographical and atmospheric conditions of the flyway that make it profitable for raptors to fly over oceans for hundreds of kilometres. Further studies using high-resolution tracking complemented with direct observations are needed to better understand the influence of islands on migratory behaviour of the birds with respect to stop-over and refuelling, flight type, and the use of wind and thermals. Moreover, such studies can shed light on the role that sea thermals play in raptor migration in the oceanic flyway.

## Supplementary Material

Input data for step selection function

## Data accessibility

Input data: electronic supplementary material (S1).
